# Supplementary material for: Genomic Characterization Provides an Insight into the Pathogenicity of the Poplar Canker Bacterium Lonsdalea populi
Source: Genes (Basel). 2021 Feb 9;12(2):246. doi: 10.3390/genes12020246 (PMC7914447; doi:10.3390/genes12020246)
Supplement: Supplementary file 1 [file genes-12-00246-s001.zip › Figures, Graphics, Images/Table 1.docx]

| **Table 1 Features of *L. populi* N-5-1 genome** | |
| --- | --- |
| **Features** | ***L. populi* N-5-1** |
| Genome Size | 3859707 |
| GC Content (%) | 56.85 |
| CDS^a^ | 3327 |
| CDS average Length | 917.93 |
| tRNA | 75 |
| rRNA(5S rRNA, 16S rRNA, 23S rRNA) | 22 |
| Other ncRNA | 10 |
