# Supplementary material for: Genomic Characterization Provides an Insight into the Pathogenicity of the Poplar Canker Bacterium Lonsdalea populi
Source: Genes (Basel). 2021 Feb 9;12(2):246. doi: 10.3390/genes12020246 (PMC7914447; doi:10.3390/genes12020246)
Supplement: Supplementary file 1 [file genes-12-00246-s001.zip › Figures, Graphics, Images/Table 2.docx]

| **Table 2 Summary of putative TCS proteins in *L.populi* N-5-1** | | | |
| --- | --- | --- | --- |
| **Gene module** | **HK/RR** | **Gene module** |  |
| **TCS** | GL000251/GL000250 | **Hybrid HK** | GL000625 |
|  | GL000776/GL000777 |  | GL001047 |
|  | GL000952/GL000951 |  | GL003222 |
|  | GL001049/GL001048 | **Orphan HK** | GL000004 |
|  | GL001140/GL001139 |  | GL000382 |
|  | GL001547/GL001548 |  | GL001027 |
|  | GL001580/GL001581 |  | GL001307 |
|  | GL001782/GL001781 |  | GL001885 |
|  | GL001852/GL001851 |  | GL001890 |
|  | GL001994/GL001993 |  | GL002494 |
|  | GL002654/GL002655 |  | GL003203 |
|  | GL002081/GL002080 | **Orphan RR** | GL000423 |
|  | GL003100/GL003101 |  | GL001261 |
|  | GL003373/GL003372 |  | GL001275 |
|  | GL003423/GL003422 |  | GL001311 |
|  | GL003452/GL003453 |  | GL001312 |
|  | GL003474/GL003473 |  | GL001708 |
|  |  |  | GL002169 |
|  |  |  | GL002492 |
