# Supplementary material for: Genomic Characterization Provides an Insight into the Pathogenicity of the Poplar Canker Bacterium Lonsdalea populi
Source: Genes (Basel). 2021 Feb 9;12(2):246. doi: 10.3390/genes12020246 (PMC7914447; doi:10.3390/genes12020246)
Supplement: Supplementary file 1 [file genes-12-00246-s001.zip › Figures, Graphics, Images/Table 3.docx]

| **Table 3 General genome characters of the** [**twelve**](C:/Users/%E6%99%93%E8%90%8C/AppData/Local/youdao/dict/Application/8.9.3.0/resultui/html/index.html#/javascript:;) **associated strains used in this study** | | | | | | |
| --- | --- | --- | --- | --- | --- | --- |
| **Strains** | **No. of NCBI accession** | **Genome size（bp）** | **CDSs** | **GC%** | **rRNA** | **tRNA** |
| *L. populi* N-5-1 | NZ_ CP065534 | 3,859,707 | 3327 | 56.85 | 22 | 75 |
| *L.populi*.HEZEL.2.1.2 | NZ_RJUH01000001.1 | 3,718,244 | 3100 | 55.4 | 10 | 68 |
| *L.populi.*L2-3 | NZ_RJUI01000001.1 | 3,651,504 | 3046 | 55.4 | 12 | 68 |
| *L.populi.*CFCC13097 | NZ_LUSU01000001.1 | 3,686,134 | 3068 | 55.3 | 4 | 61 |
| *L.britannica*.477 | NZ_CP023009.1 | 4,015,569 | 3348 | 55.1 | 22 | 83 |
| *L.quercina* ATCC29281 | NZ_FNQS01000023.1 | 3,779,259 | 3188 | 55.6 | 12 | 61 |
| *L.iberica.*LMG26264 | NZ_LUTP01000001.1 | 3,781,823 | 3081 | 55 | 5 | 48 |
| *D.chrysanthemi*.Ech1591 | NC_012912.1 | 4,813,854 | 4098 | 54.5 | 22 | 74 |
| *B.nigrifluens*.DSM.30175 | NZ_CP034036.1 | 4,891,702 | 4550 | 55.9 | 22 | 72 |
| *D.paradisiaca*.NCPPB.2511 | NZ_CM001857.1 | 4,631,867 | 3775 | 55 | 22 | 74 |
| *B.alni.*NCPPB.3934 | NZ_MJLZ01000001.1 | 4,126,956 | 3768 | 51 | 13 | 65 |
| *E.pyrifoliae.*Ep1.96 | NC_012214.1 | 4,072,846 | 3502 | 53.4 | 22 | 75 |
| *E.amylovora*.CFBP1430 | NC_013961.1 | 3,833,832 | 3356 | 53.6 | 22 | 77 |
