# Supplementary material for: Genomic Characterization Provides an Insight into the Pathogenicity of the Poplar Canker Bacterium Lonsdalea populi
Source: Genes (Basel). 2021 Feb 9;12(2):246. doi: 10.3390/genes12020246 (PMC7914447; doi:10.3390/genes12020246)
Supplement: Supplementary file 1 [file genes-12-00246-s001.zip › Figures, Graphics, Images/Table 4.docx]

| **Table  4  Distribution of mobile genetic elements in N-5-1 genome** | | |
| --- | --- | --- |
| **Strain** | **N.O of GeneIsland** | **N.O of Prophage** |
| *L. populi* N-5-1 | 41 | 7 |
| *B.nigrifluens*.DSM.30175 | 21 | 11 |
| *E.amylovora*.CFBP1430 | 23 | 4 |
| *L.britannica*.477 | 8 | 6 |
