# Supplementary material for: Genomic Characterization Provides an Insight into the Pathogenicity of the Poplar Canker Bacterium Lonsdalea populi
Source: Genes (Basel). 2021 Feb 9;12(2):246. doi: 10.3390/genes12020246 (PMC7914447; doi:10.3390/genes12020246)
Supplement: Supplementary file 1 [file genes-12-00246-s001.zip › Figures, Graphics, Images/Table S2.docx]

| **Table S2 Genes with a potential role in antibiotic resistance of *L. populi*N-5-1** | | | | | | |
| --- | --- | --- | --- | --- | --- | --- |
| **Genes’ ID** | **Resistance Type** | **Antibiotic_Resistance** | **Genes’ ID** | | **Resistance Type** | **Antibiotic_Resistance** |
| N-5-1GL000259 | *pbp1a* | Penicillin | N-5-1GL001034 | | *rosb* | Fosmidomycin |
| N-5-1GL000456 | *dfra12* | Trimethoprim | N-5-1GL001106 | | *pbp2* | Penicillin |
| N-5-1GL000459 | *ksga* | Kasugamycin | N-5-1GL001124 | | *emre* | Aminoglycoside |
| N-5-1GL000613 | *baca* | Bacitracin | N-5-1GL001238 | | smec | Fluoroquinolone |
| N-5-1GL000711 | *tet34* | Tetracycline | N-5-1GL001239 | | acrb | Aminoglycoside,Glycylcycline,Macrolide,Beta_lactam,Acriflavin |
| N-5-1GL001015 | *acrb* | minoglycoside,Glycylcycline,Macrolide,Beta_lactam,Acriflavin | N-5-1GL001240 | | acra | Aminoglycoside,Glycylcycline,Macrolide,Beta_lactam,Acriflavin |
| N-5-1GL001016 | *acra* | Aminoglycoside,Glycylcycline,Macrolide,Beta_lactam,Acriflavin | N-5-1GL001473 | | mdtk | Enoxacin,norfloxacin |
| N-5-1GL002556 | pbp1b | penicillin | N-5-1GL001841 | | mdth | deoxycholate,fosfomycin |
| N-5-1GL002638 | mdtg | deoxycholate,fosfomycin | N-5-1GL001988 | bcr | |  |
| N-5-1GL002657 | macb | Macrolide | N-5-1GL001473 | mdtk | | Enoxacin,norfloxacin |
| N-5-1GL003208 | tolc | Aminoglycoside,Glycylcycline,Macrolide,Beta_lactam,Acriflavin | N-5-1GL001240 | acra | | Aminoglycoside,Glycylcycline,Macrolide,Beta_lactam,Acriflavin |
| N-5-1GL001841 | mdth | deoxycholate,fosfomycin | N-5-1GL002556 | pbp1b | | penicillin |
| N-5-1GL001988 | bcr | - | N-5-1GL002638 | mdtg | | deoxycholate,fosfomycin |
