# Supplementary material for: Genomic Characterization Provides an Insight into the Pathogenicity of the Poplar Canker Bacterium Lonsdalea populi
Source: Genes (Basel). 2021 Feb 9;12(2):246. doi: 10.3390/genes12020246 (PMC7914447; doi:10.3390/genes12020246)
Supplement: Supplementary file 1 [file genes-12-00246-s001.zip › Figures, Graphics, Images/Table S3.docx]

| **Table S3 Predict the similarity of gene clusters to known gene clusters in the four associated strains** | | | | | |
| --- | --- | --- | --- | --- | --- |
| **Strain** | **No. of NCBI accession** | **Cluster** | **Most similar known cluster** | **Similarity** | **MIBiG accession** |
| *L. populi* N-5-1 | NZ_ CP065534 | Cluster 3 | rhizomide A / B / C | 100% | BGC0001758 |
|  |  | Cluster 4 | bicornutin A1 /A2 | 100% | BGC0001135 |
|  |  | Cluster 5 | rhizomide A / B / C | 100% | BGC0001758 |
|  |  | Cluster 6 | luminmide | 100% | BGC0001128 |
| *E.amylovora*.CFBP1430 | NC_013961.1 | Cluster 19 | rhizomide A / B / C | 100% | BGC0001758 |
| *L.britannica*.477 | NZ_CP023009.1 | Cluster 23 | rhizomide A / B / C | 100% | BGC0001758 |
|  |  | Cluster 24 | rhizomide A / B / C | 100% | BGC0001758 |
|  |  | Cluster 25 | xenotetrapeptide | 100% | BGC0001132 |
|  |  | Cluster 26 | aculleximycin | 17% | BGC0000002 |
