# Supplementary material for: Genomic Characterization Provides an Insight into the Pathogenicity of the Poplar Canker Bacterium Lonsdalea populi
Source: Genes (Basel). 2021 Feb 9;12(2):246. doi: 10.3390/genes12020246 (PMC7914447; doi:10.3390/genes12020246)
Supplement: Supplementary file 1 [file genes-12-00246-s001.zip › Figures, Graphics, Images/Table S4.docx]

| **Table S4** **Secreted signal peptide length and corresponding protein** | | | |
| --- | --- | --- | --- |
| **Secreted protein** | **Length (bp)** | **Secreted protein** | **Length(bp)** |
| GL000261 | 177 | GL001614 | 115 |
| GL000381 | 566 | GL001622 | 236 |
| GL000390 | 457 | GL001636 | 195 |
| GL000537 | 253 | GL001843 | 187 |
| GL000551 | 83 | GL002188 | 233 |
| GL000567 | 42 | GL002241 | 119 |
| GL000787 | 115 | GL002489 | 385 |
| GL000859 | 358 | GL002728 | 44 |
| GL000885 | 399 | GL002789 | 317 |
| GL000886 | 383 | GL002998 | 185 |
| GL001104 | 342 | GL002999 | 176 |
| GL001185 | 140 | GL003061 | 370 |
| GL001428 | 187 | GL003209 | 237 |
