# Supplementary material for: Genomic Characterization Provides an Insight into the Pathogenicity of the Poplar Canker Bacterium Lonsdalea populi
Source: Genes (Basel). 2021 Feb 9;12(2):246. doi: 10.3390/genes12020246 (PMC7914447; doi:10.3390/genes12020246)
Supplement: Supplementary file 1 [file genes-12-00246-s001.zip › Figures, Graphics, Images/Table S5.docx]

| **Table S5 N-5-1 and other 12 strains genome ANI^a^** | | | | | | | | | | | | | | |
| --- | --- | --- | --- | --- | --- | --- | --- | --- | --- | --- | --- | --- | --- | --- |
| **Strain** | **NO.** | **1** | **2** | **3** | **4** | **5** | **6** | **7** | **8** | **9** | **10** | **11** | **12** | **13** |
| *L. populi* N-5-1 | **1** |  | 74.93 | 76.46 | 76.29 | 75.76 | 72.08 | 72.17 | 90.37 | 89.39 | **99.30** | **99.36** | **99.35** | 89.67 |
| *B.alni.*NCPPB.3934 | **2** | 74.30 |  | 86.64 | 75.40 | 75.03 | 72.41 | 72.55 | 74.69 | 74.50 | 74.78 | 74.82 | 74.88 | 74.60 |
| *B.nigrifluens*.DSM.30175 | **3** | 76.05 | 84.34 |  | 76.06 | 75.65 | 73.03 | 72.53 | 75.87 | 75.58 | 76.31 | 76.33 | 75.92 | 75.73 |
| *D.chrysanthemi*.Ech1591 | **4** | 76.19 | 75.29 | 76.25 |  | 79.02 | 72.48 |  | 76.05 | 75.88 | 76.05 | 76.06 | 75.92 | 75.73 |
| *D.paradisiaca*.NCPPB.2511 | **5** | 75.49 | 74.92 | 75.80 | 78.90 |  | 72.05 | 72.18 | 75.53 | 75.45 | 75.43 | 75.43 | 75.44 | 75.46 |
| *E.amylovora*.CFBP1430 | **6** | 72.29 | 72.47 | 73.40 | 72.61 | 72.32 |  | 91.25 | 72.35 | 72.22 | 72.31 | 72.31 | 72.32 | 72.23 |
| *E.pyrifoliae.*Ep1.96 | **7** | 72.45 | 72.54 | 73.37 | 72.79 | 72.36 | 91.28 |  | 72.57 | 72.54 | 72.36 | 72.43 | 72.49 | 72.35 |
| *L.britannica*.477 | **8** | 90.70 | 74.93 | 76.22 | 76.18 | 75.78 | 72.30 | 72.49 |  | 90.06 | 90.70 | 90.77 | 90.80 | 89.51 |
| *L.iberica.*LMG26264 | **9** | 89.77 | 74.78 | 76.01 | 76.08 | 75.79 | 72.30 | 72.32 | 90.06 |  | 89.77 | 89.78 | 89.91 | 92.28 |
| *L.populi.*CFCC13097 | **10** | **99.43** | 74.91 | 76.78 | 76.18 | 75.77 | 72.20 | 72.18 | 90.59 | 89.59 |  | **99.98** | **99.81** | 89.95 |
| *L.populi*.HEZEL.2.1.2 | **11** | **99.43** | 74.99 | 76.84 | 76.20 | 75.76 | 72.16 | 72.15 | 90.55 | 89.54 | **99.93** |  | **99.80** | 89.90 |
| *L.populi.*L2-3 | **12** | **99.43** | 75.07 | 76.40 | 76.23 | 75.73 | 72.18 | 72.22 | 90.60 | 89.57 | **99.78** | **99.83** |  | 89.90 |
| *L.quercina.*ATCC29281 | **13** | 90.01 | 74.79 | 76.18 | 76.13 | 75.77 | 72.19 | 72.20 | 89.53 | 92.26 | 90.03 | 90.06 | 90.12 |  |

^a^ANI:Average nucleotide identity
