# Supplementary figures and images for: Genomic Characterization Provides an Insight into the Pathogenicity of the Poplar Canker Bacterium Lonsdalea populi
Source: Genes (Basel). 2021 Feb 9;12(2):246. doi: 10.3390/genes12020246 (PMC7914447; doi:10.3390/genes12020246)

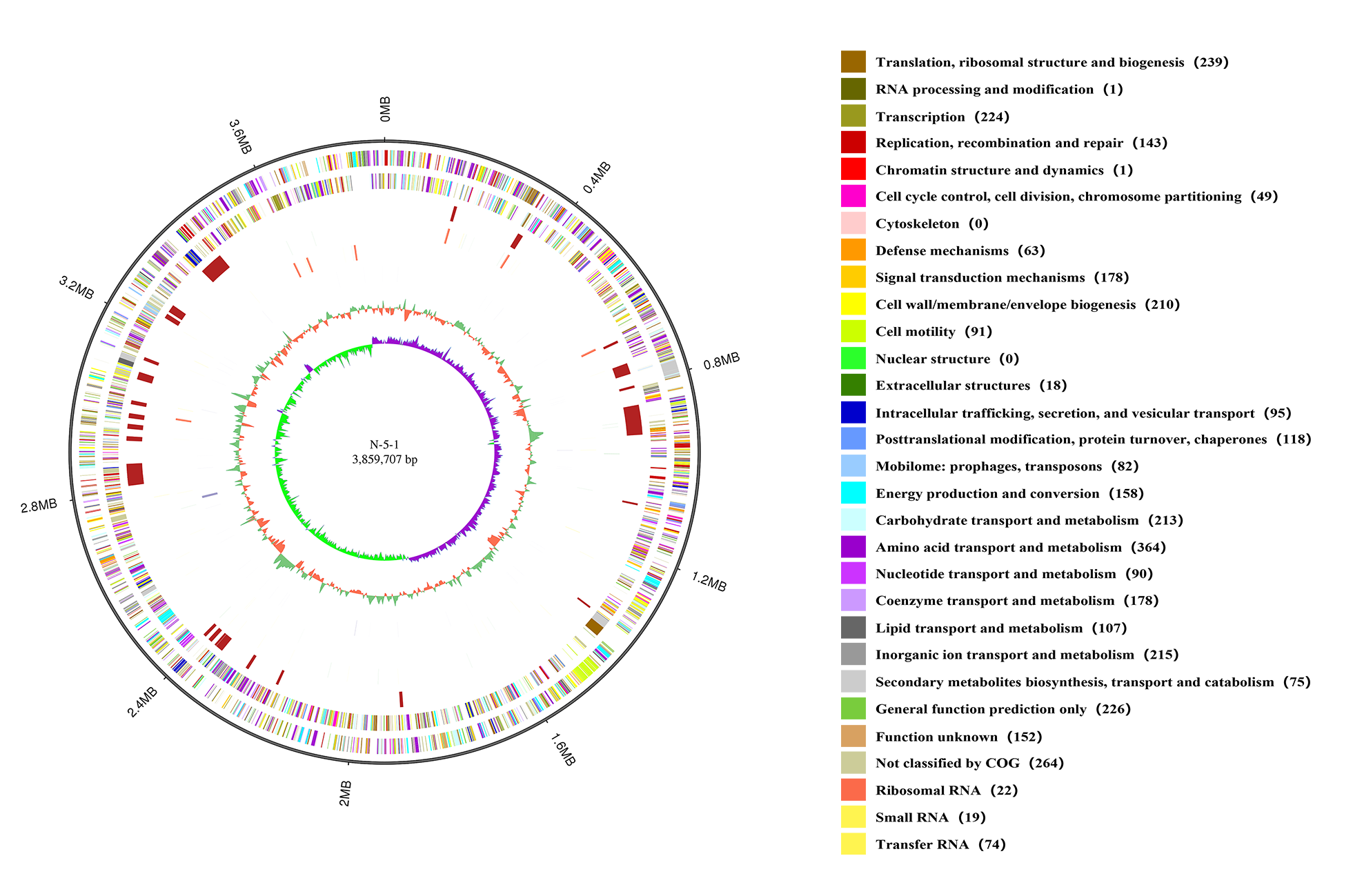

Supplement: Supplementary file 1 [file genes-12-00246-s001.zip › Figures, Graphics, Images/Fig 1.tif]

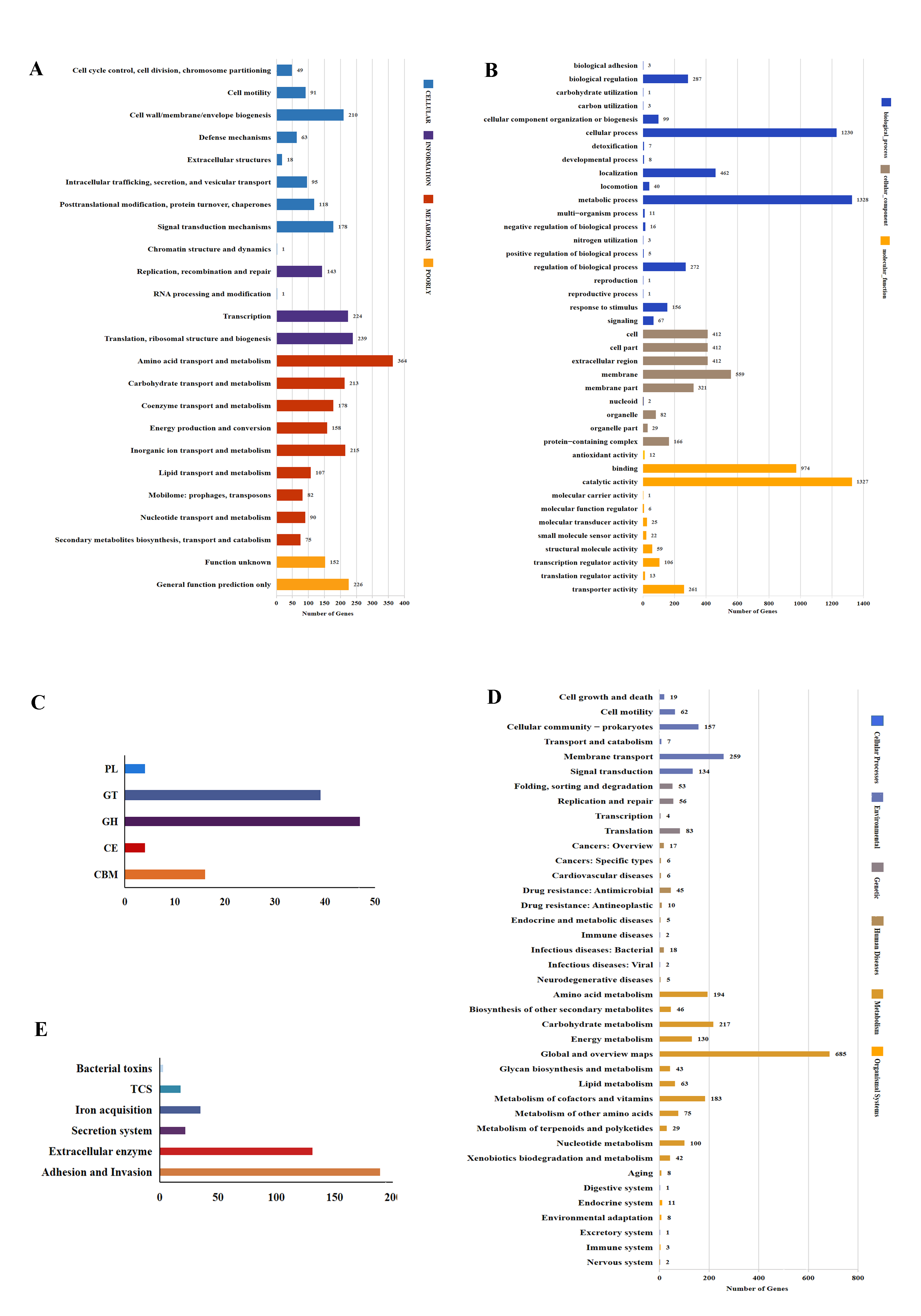

Supplement: Supplementary file 1 [file genes-12-00246-s001.zip › Figures, Graphics, Images/Fig 2.tif]

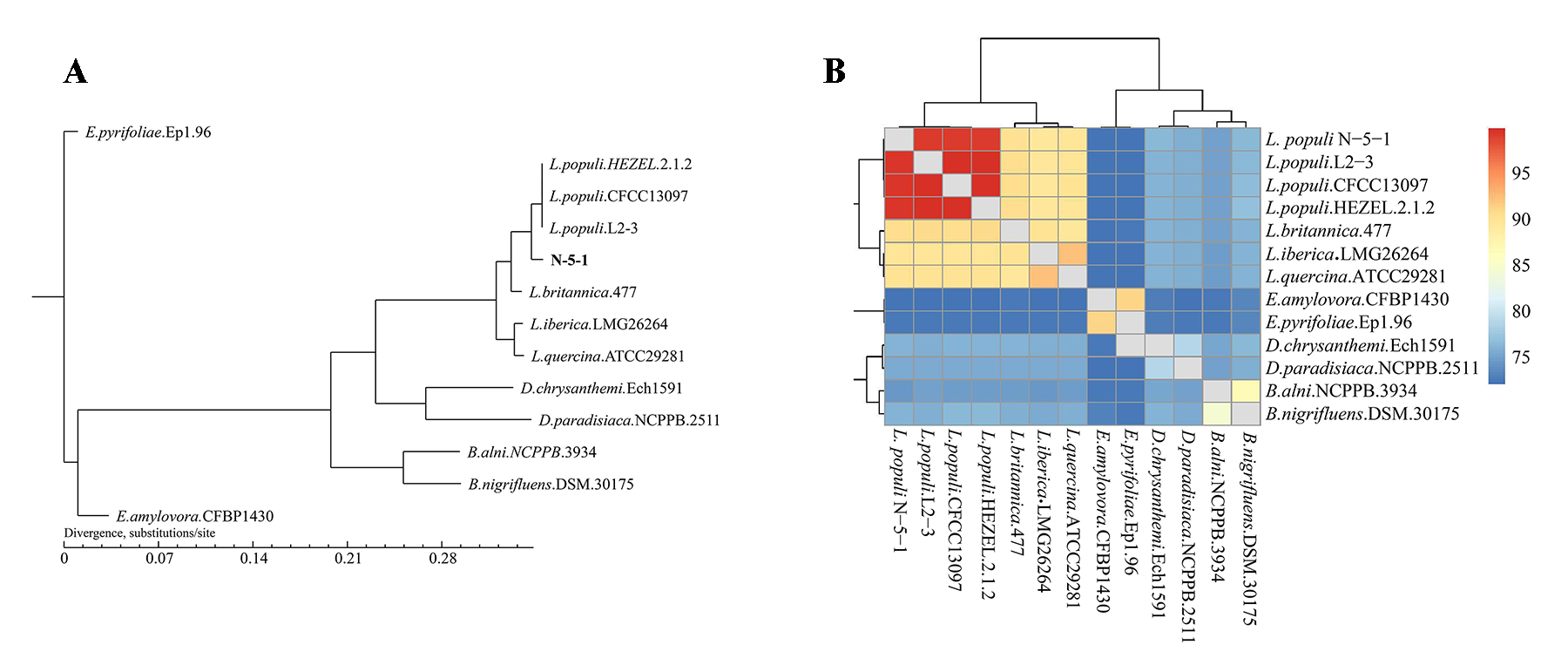

Supplement: Supplementary file 1 [file genes-12-00246-s001.zip › Figures, Graphics, Images/Fig 4.tif]

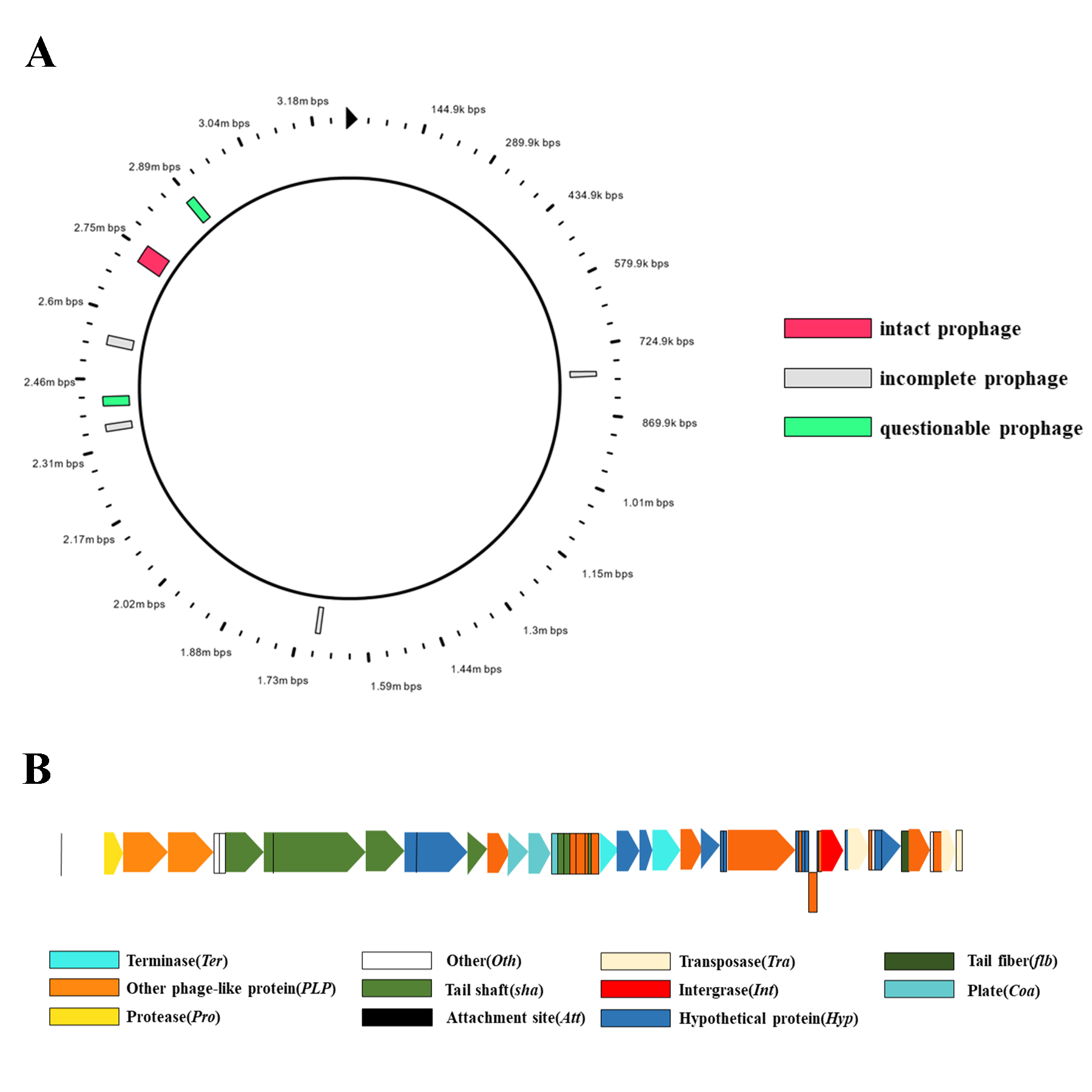

Supplement: Supplementary file 1 [file genes-12-00246-s001.zip › Figures, Graphics, Images/Fig 5.tif]

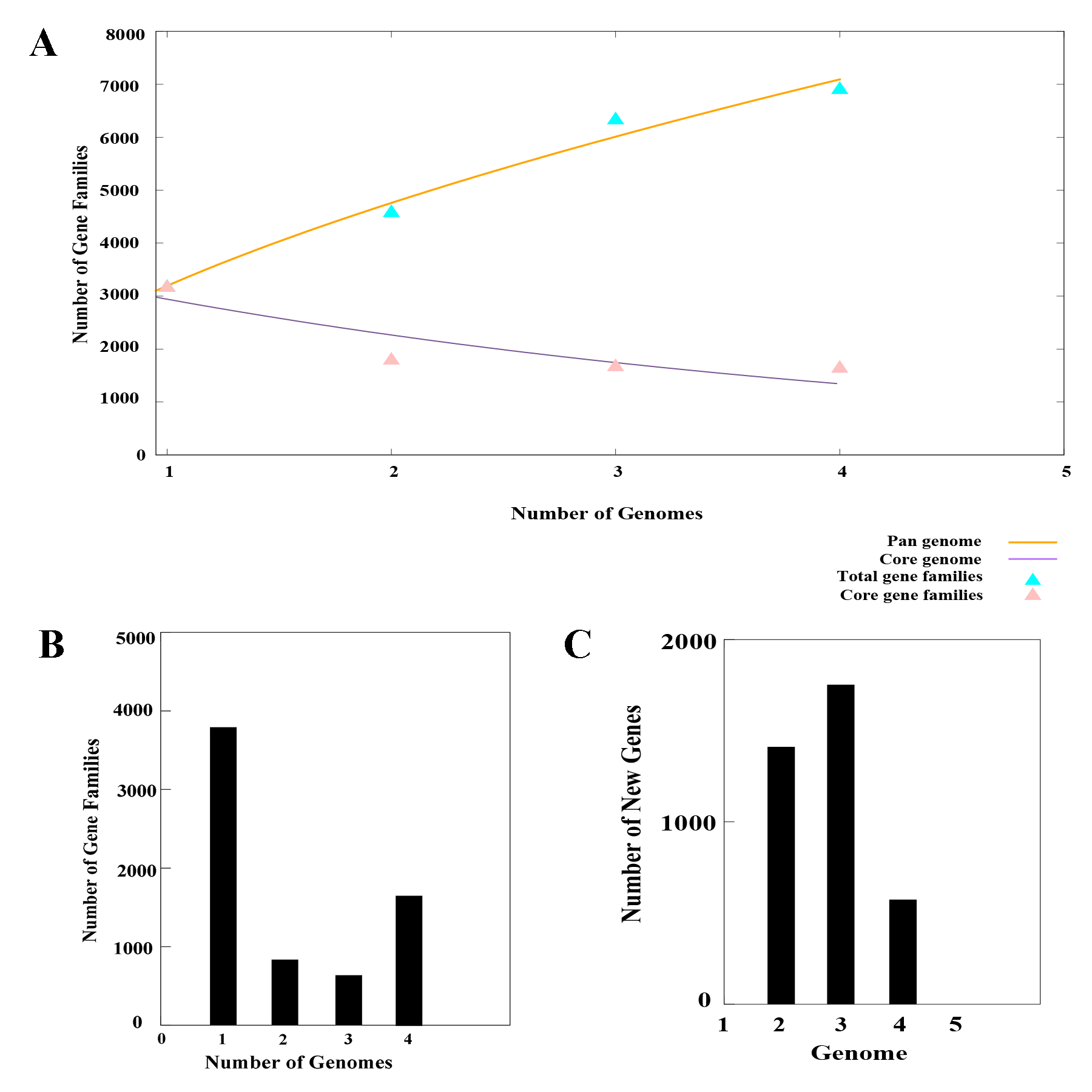

Supplement: Supplementary file 1 [file genes-12-00246-s001.zip › Figures, Graphics, Images/Fig 6.tif]

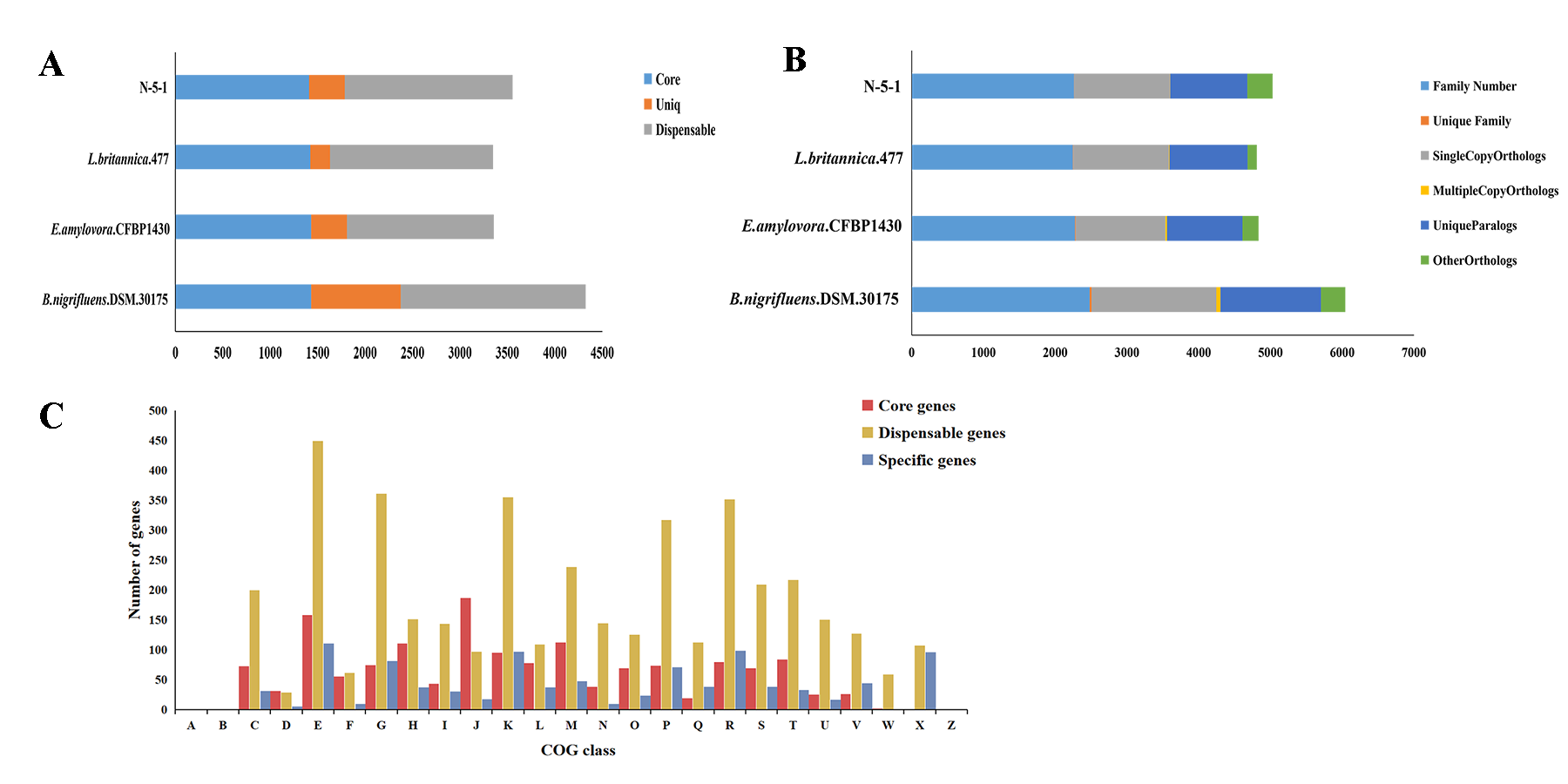

Supplement: Supplementary file 1 [file genes-12-00246-s001.zip › Figures, Graphics, Images/Fig 7.tif]

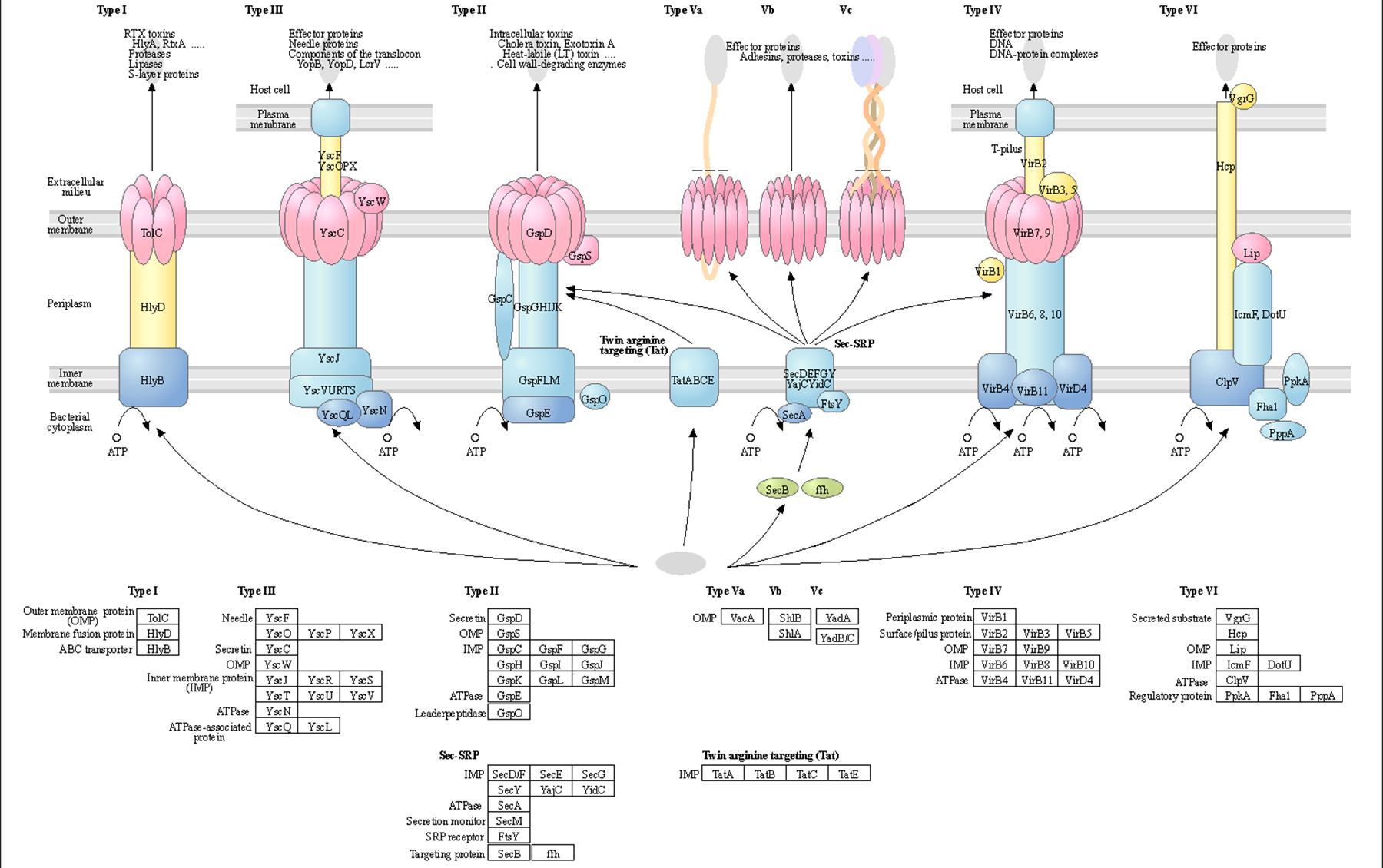

Supplement: Supplementary file 1 [file genes-12-00246-s001.zip › Figures, Graphics, Images/Fig S1.tif]

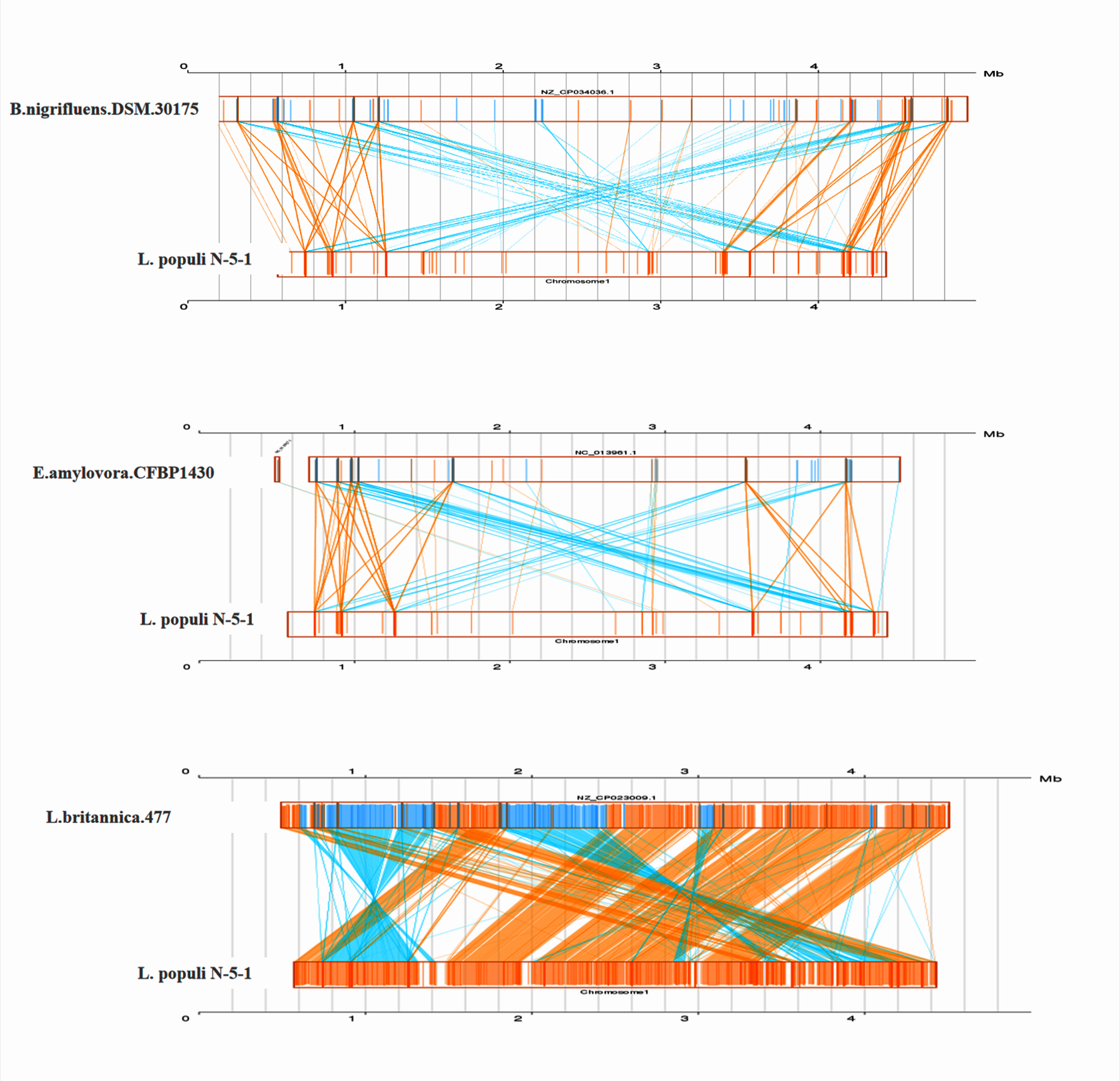

Supplement: Supplementary file 1 [file genes-12-00246-s001.zip › Figures, Graphics, Images/Fig S2.tif]

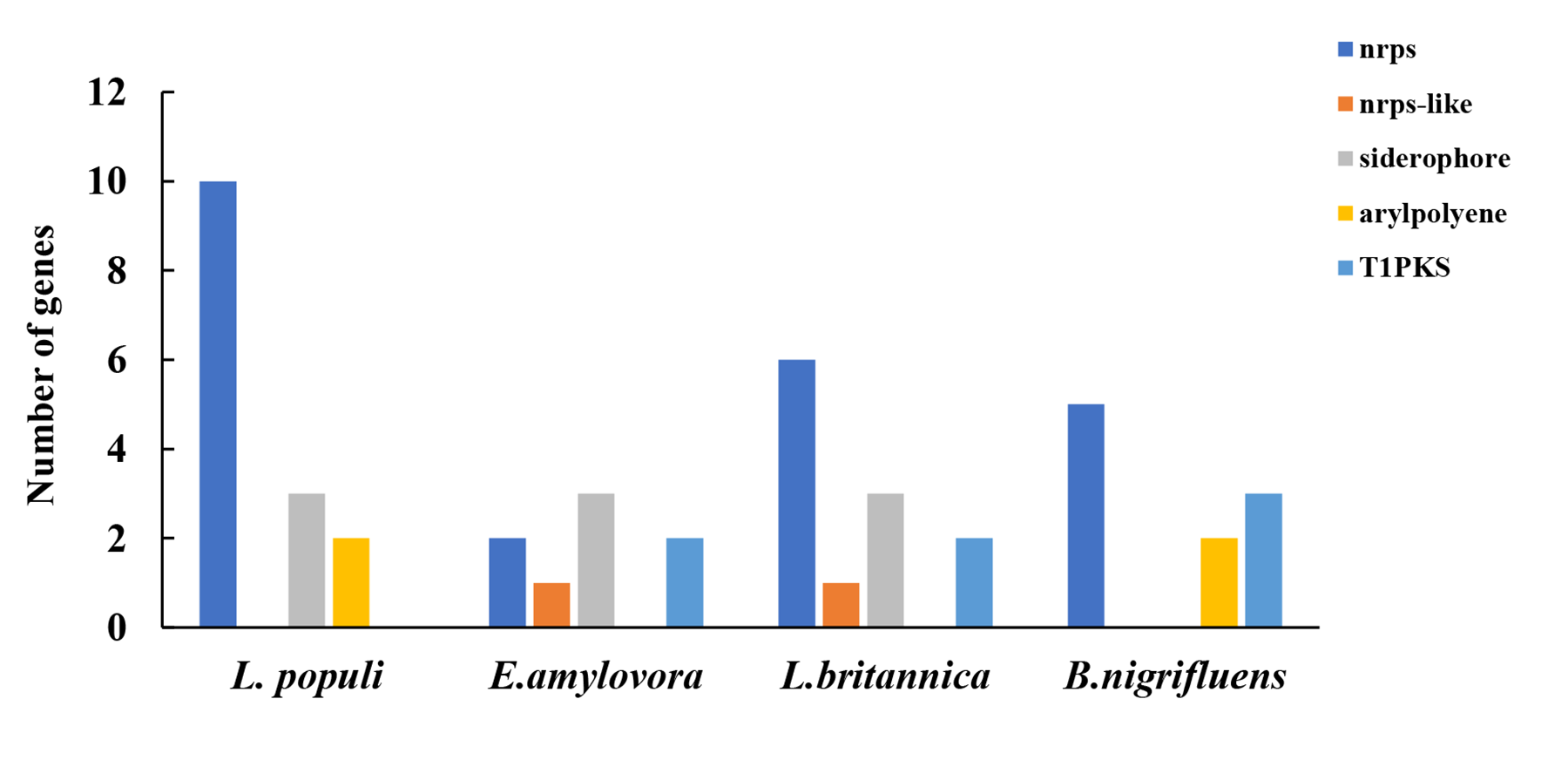

Supplement: Supplementary file 1 [file genes-12-00246-s001.zip › Figures, Graphics, Images/Fig 3.tif]
